# Levels of Telehealth Use, Perceived Usefulness, and Ease of Use in Behavioral Healthcare Organizations After the COVID-19 Pandemic

**DOI:** 10.1007/s11414-024-09902-6

**Published:** 2024-08-07

**Authors:** Kathryn Fleddermann, Lydia Chwastiak, Ashley Fortier, Heather Gotham, Ann Murphy, Rachel Navarro, Stephanie Tapscott, Todd Molfenter

**Affiliations:** 1 Center for Health Enhancement Systems Studies, Department of Industrial and Systems Engineering, University of Wisconsin-Madison, Madison, WI USA; 2 Department of Psychiatry and Behavioral Sciences, University of Washington School of Medicine, Seattle, WA USA; 3 Western Interstate Commission for Higher Education (WICHE), Boulder, CO USA; 4 Department of Psychiatry and Behavioral Sciences, Stanford University School of Medicine, Stanford, CA USA; 5 Department of Psychiatric Rehabilitation and Counseling Professions, Rutgers University, New Brunswick, NJ USA; 6 Department of Education, Health, and Behavior Studies, Counseling Psychology Ph.D. Program, University of North Dakota, Grand Forks, ND USA; 7 Department of Health and Policy Management, Rollins School of Public Health, Emory University, Atlanta, GA USA

## Abstract

The use of telehealth in behavioral healthcare increased significantly since the start of the COVID-19 pandemic and remains high even as a return to in-person care is now feasible. The use of telehealth is a promising strategy to increase access to behavioral healthcare for underserved and all populations. Identifying opportunities to improve the provision of telehealth is vital to ensuring access. An online survey about the current use of, and attitudes toward, telehealth was conducted by five Mental Health Technology Transfer Center (MHTTC) regional centers and the MHTTC Network Coordinating Office. The national MHTTC network provides training and technical assistance, to support the behavioral health workforce to implement evidence-based treatments. Three hundred and sixty-five respondents from 43 states and Puerto Rico participated. The majority of respondents were clinical providers (69.3%). Nearly all (*n* = 311) respondents reported providing at least one telehealth service at their organization, but the number and type of services varied substantially. Respondents had positive views of both video-based and phone-based services, but most had some preference for video-based telehealth services. Other services, including text message reminders, medication services, and mobile apps for treatment or recovery, were offered via telehealth by ~ 50% or fewer of respondents’ organizations. Many organizations have areas where they could expand their telehealth use, allowing them to extend the reach of their services and increase access for populations that experience barriers to service access, though organizational barriers may still prevent this.

## Introduction

At the onset of the COVID-19 pandemic in the United States, many healthcare organizations were underprepared for the sudden transition to telehealth care that was required. However, the conditions of the pandemic necessitated rapid shifts in the provision of healthcare, and organizations rose to the challenge to continue to meet the needs of their patients.^1,2^ In the current phase of the pandemic, telehealth care is no longer a necessity, but many providers and patients continue to prefer this method of care.^3,4^ Understanding the state of telehealth care within behavioral healthcare organizations will allow organizations to continue to provide this service as efficiently and effectively as possible.^5–7^

The pandemic led to a massive increase in the number of telehealth services that many healthcare organizations provided, as patients were unable to come to in-person appointments due to stay-at-home orders.^2,8,9^ Providers reported conducting more than six times as many telehealth visits in the months immediately after March 2020, with many shifting from less than 1% of visits a day on telehealth to over 70%.^10,11^ Even as the regulations and restrictions of the pandemic began to ease, rates of telehealth across medical specialties remained high with ~ 25% of patients receiving some services via telehealth between 2021 and 2022.^10,12^ While there has been some decline in overall telehealth visits over the course of the pandemic and into more recent times, when restrictions on healthcare options have been fully eliminated, much of this decline was driven by an increased return to in-person visits for physical health conditions. Telehealth use for mental health conditions has remained much higher, with roughly 30–40% of visits being completed by telehealth.^13,14^ Though these rates have also lessened from the initial peak in mid-2020, they have shown a slower decline and remain much higher than rates for other areas of healthcare.

Compared to in-person care, telehealth increases access to healthcare services, but the provision of behavioral healthcare presents unique challenges for telehealth. Many behavioral healthcare providers find the provision of services via telehealth more challenging because of less personal connection with clients and because of regulations and restrictions for some types of services, such as medication for alcohol and opioid use disorders (MAUD and MOUD).^15–17^ However, telehealth lowers barriers to care for many clients who have been unable to access care because of logistical barriers or stigma; telehealth presents an opportunity to increase the number of individuals receiving care.^18^ The ongoing need for telehealth options for behavioral healthcare is clear, as the rate of telehealth use in behavioral health organizations has remained higher than in other medical settings, even as the pandemic wanes.^13,14^

In order for telehealth to become a regular option in behavioral healthcare, many organizations will need ongoing shifts in their operations in order to better support the use of telehealth. Increasing training to promote experience and comfort using the necessary technology and having motivated “champions” for telehealth use within an organization who can encourage and support other providers in their use of the technology can both help healthcare organizations facilitate the use of telehealth.^19^ Additionally, telehealth use is more likely to be continued if viewed positively by patients, as telehealth has been shown to reduce costs and increase access for many populations, including those who have been historically underserved.^20^ Taken together, understanding more about what supports, at the organizational, provider, and patient level, are most common within behavioral healthcare organizations is important to understanding the landscape of telehealth care and how to sustain or promote its use within organizations if desired.

As organizations move into a new era of providing care to clients, likely combining in-person and virtual care to best meet the needs of clients, having a clearer picture of the state of telehealth within behavioral health organizations is important to better prepare for future steps. This study reports the findings of an online survey of a large sample of behavioral health professionals (psychologists, counselors, social workers, case managers, administrators, etc.) across care settings and geographic locations. The study aimed to inform a deeper understanding of how telehealth is currently being utilized in behavioral healthcare settings and to compare views of different types of telehealth services. To this end, the authors seek to describe current levels of usage of a variety of telehealth service types in behavioral health organizations and compare the frequency of use of in-person, telephone, and video service use across services. Further, the authors looked to compare the perceived usefulness and ease of use of telephone and video counseling in behavioral health organizations and respondent views of existing levels of support, at the organizational, provider, and patient level, for the use of telehealth services.

## Methods

### Respondents

Individuals employed in administrative and clinical roles at behavioral healthcare organizations were asked to complete a self-administered survey online in REDCap between September and December 2022.

### Procedures

A link to an online survey was distributed by five regional Mental Health Technology Transfer Centers (MHTTC) and the Network Coordinating Office. Emails were sent to Centers’ listservs and were distributed during trainings to behavioral healthcare organizations. The MHTTC Network, funded by the Substance Abuse and Mental Health Services Administration (SAMHSA), consists of ten regional centers and a Network Coordinating Office which provide technical assistance and training to accelerate the implementation of evidence-based mental health prevention, treatment, and recovery supports. Each regional center serves the states and territories of one of the US Department of Health and Human Services–designated regions. The MHTTCs have strong connections with local behavioral health organizations, and the Network Coordinating Office has a large national listserv, and these were used to disseminate the survey.

The survey was distributed by only five out of ten MHTTCs due to existing connections in the research group, which allowed for more efficient distribution. Because the MHTTCs have strong reach both within and outside of their service areas and there is a national listserv, national distribution occurred. Individuals can sign up to be on MHTTC listservs directly from the MHTTC website if they are interested in receiving training opportunities and content. Additionally, many individuals on these lists have attended previous MHTTC trainings and are on the lists from those services. Surveys were sent via newsletter and email lists, and recipients were encouraged to pass the survey on to others not on the original list who might be interested in participating. Respondents were not paid or otherwise incentivized to complete the survey.

This study was reviewed and approved by the University of Wisconsin-Madison Institutional Review Board (protocol # 2020–0551). The STROBE reporting guidelines were followed for this manuscript.^21^

### Measures

The survey used for this project was based on a previous survey that assessed telehealth use during the COVID-19 pandemic.^22^ Included measures and a description of what they assessed are in Appendix 1. The goal of this survey was to gain similar information in a post-pandemic timeframe.

#### Demographics

General demographic information was collected about respondents’ organization and roles, including organization name and location (state and zip code), what type of area the organization is located in, if the organization primarily serves tribal communities, and the participant’s role in their organization. A full list of demographic information collected is included in Appendix 1. Because only a small number of respondents selected that their organization is located in a suburban area or small city, respondents’ community type was recategorized as either rural or urban by using USDA rural–urban commuting area codes, which give each zip code a rating from 1 to 10.^23^ Based on HRSA rural health definitions, any zip code rated 4–10 was recategorized as rural while the rest were recategorized as urban.^24^ Provider type was recategorized into administrator (administrator, clinical supervisor), clinician (counselor, social worker, psychologist), medical provider (physician/prescriber, nurse), and other (case worker, peer support worker, other).

#### Provision of Telehealth Services

To assess current levels of telehealth service provision across a variety of potential service types, respondents were asked whether their organization currently utilized a variety of possible telehealth services and whether they provided those services in-person, via telephone, and/or via video. Telehealth services selected to be assessed were based on the previous survey completed.^22^ Telehealth services asked about are listed in Appendix 1.

#### Organizational Readiness for Technology Use

To assess organizational readiness for the utilization of telephone and video technology in practice, the Organizational Readiness for Technology Use predictive tool developed by Gustafson et al. was used.^25^ This tool asks respondents to rate current supports and readiness in their organization for the use of both phone and video technology tools. The types of supports asked about and scales used are listed in Appendix 1.

#### Usefulness and Ease of Use

To assess the perceived usefulness and ease of use of phone and video technologies, this survey included scales from the Technology Acceptance Model (TAM).^26,27^ This is a brief scale that asks about a variety of aspects of usefulness and ease of use to better understand what may be supporting or preventing use. A full list of questions is included in Appendix 1.

### Analysis

Frequencies of demographic information and organization characteristics were calculated using SPSS. Some variables were recategorized to create fewer categories for analysis. All respondents who advanced in the survey beyond demographic questions were included in analyses, even if they did not respond to some questions. Responses were not required for any survey items, resulting in smaller sample sizes for some analyses. This is indicated where applicable.

Means and standard deviations were calculated for all questions. On technology use questions, because respondents could indicate “yes” to more than one of the modalities, a related-samples Cochran’s Q test was run to assess differences in frequencies of service delivery in-person vs. by telephone vs. by video. A Bonferroni correction was completed on the pairwise comparisons to correct for the effects of multiple comparisons. To assess whether there are differences in views of and supports available for telephone vs. video-based telehealth in organizations, paired samples *t*-tests were run for each question. Further, to assess group differences in views of usefulness and ease of use according to respondent role, independent sample *t*-tests were run.

## Results

### Respondent Characteristics

Out of 480 individuals who started the survey, 365 advanced past the demographics questions and were included in analyses. Respondents were from 43 states and Puerto Rico, with the largest number of participants from Michigan (*n* = 73), Illinois (*n* = 58), and Wisconsin (*n* = 39). A summary of respondent characteristics is provided in Table 1. Respondent characteristics remained similar for analyses when smaller sample sizes were utilized, with similar proportions of each characteristic represented.

**Table 1:** Summary of respondent characteristics (*N* = 365)

	Number	Percentage
Organization location		
Urban Rural	270 93	74.0 25.5
Tribal community		
Yes No	20 344	5.5 94.2
Organization type		
Health system Federally Qualified Health Center (FQHC) Specialty behavioral health provider: 6 + sites Specialty behavioral health provider: 2–5 sites Specialty behavioral health provider: stand-alone site Private practice	67 11 73 83 54 68	18.4 3.0 20.0 22.7 14.8 18.6
Role		
Administrator Clinician Medical provider	60 253 50	16.4 69.3 13.8

Due to some missing data, the total ns per item ranges from 356 to 364

### Technology Use in Behavioral Health Organizations

**Figure 1:**
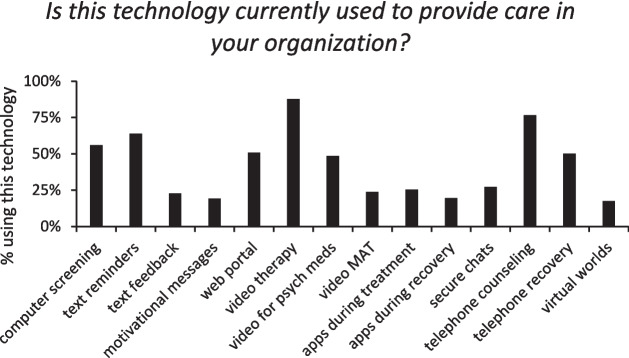
Graph of the number of respondents indicating their organization provides each type of telehealth service

The majority of respondents (85.2%) indicated their organization provided at least one service via telehealth (phone and/or video), but technology use varied widely. The most common telehealth services were video-based counseling and telephone-based counseling, with more than 75% of respondents indicating that these technologies were used in their organizations. Other commonly used technologies included text appointment reminders, computerized screenings and assessments, organizational web portals, and telephone-based recovery supports. On the other hand, the least commonly used technologies selected by respondents included virtual worlds for treatment, texted motivational messages, texts to solicit patient experience feedback, and video-based therapy to provide medication-assisted therapy (MAT). Percentages of respondents indicating their organization utilizes each of the technologies are included in Fig. 1.

Table 2 outlines the numbers of respondents indicating they provide each type of behavioral health service via in-person, telephone, or video. Respondents could indicate they provide services via more than one of the available modalities. Because respondents could indicate “yes” to more than one of the modalities, a related-samples Cochran’s *Q* test was run to assess differences in the frequency of use of modality type. For every type of service, there was a significant difference in frequency of use between at least some of the possible modalities (all *ps* < *0.001*).

**Table 2:** Counts and percentages of respondents indicating their organization provides each type of telehealth service by each of the possible modalities (in-person, telephone, or video)

Type of service	In-person (*N*)	By telephone (*N*)	By video (*N*)	*p* (in-person vs. telephone)	*p* (in-person vs. video)	*p* (telephone vs. video)
Screening and assessment	302	187	245	< 0.001	< 0.001	< 0.001
Individual therapy	293	211	277	< 0.001	0.318	< 0.001
Group therapy	234	61	146	< 0.001	< 0.001	< 0.001
Medication management	205	86	152	< 0.001	< 0.001	< 0.001
Therapy services during partial hospitalization	84	37	45	< 0.001	< 0.001	0.838
Therapy services during residential treatment	109	39	56	< 0.001	< 0.001	0.104
Peer support	193	137	132	< 0.001	< 0.001	1.000
Case management	240	187	172	< 0.001	< 0.001	0.268
Multi-disciplinary team-based care (e.g., Assertive Community Treatment)	142	92	96	< 0.001	< 0.001	1.000
In-home supports	151	86	89	< 0.001	< 0.001	1.000
Psychoeducation	233	162	193	< 0.001	< 0.001	0.004
Employment supports	141	97	93	< 0.001	< 0.001	1.000
Education supports	145	93	95	< 0.001	< 0.001	1.000

Respondents were able to select more than one answer for each type of service

Pairwise comparisons indicate that many services (screening, group therapy, medication management, residential treatment, and psychoeducation) show significant differences in the rate of provision between all three types of modalities. However, there was no difference in the rates at which respondents indicated providing individual therapy in-person versus by video (Bonferroni corrected *p* = 0.318), with both of these modalities being offered significantly more frequently than by telephone. Lastly, many types of treatments (partial hospitalization, peer support, case management, multi-disciplinary team-based treatment, in-home supports, employment supports, educational supports) showed no difference in rates of provision by video versus phone but had significantly higher rates of provision in-person.

### Telehealth Usefulness and Organizational Supports

For both telephonic and video-based counseling, respondents indicated generally favorable views on its use, benefits to their productivity and effectiveness in their work, and ease of learning and use with patients (Table 3). Respondents rated video-based counseling significantly more positively than telephonic counseling on questions regarding these technologies’ ability to improve performance, increase productivity, enhance effectiveness, and be useful (all *ps* < *0.001*). Ratings for the ease of getting video-based counseling to do what respondents wanted it to do were also significantly higher than for telephonic counseling, *p* = *0.015*. Views on usefulness and ease of use of both types of telehealth tools did not vary significantly by respondent role (administrator vs. clinician), with the exception of clinician respondents agreeing more strongly that these telehealth tools do not require a lot of effort to offer to patients.

**Table 3:** Comparisons between telephonic and video counseling on usefulness and ease-of-use scores

	Number	Telephonic counseling		Video counseling		*p*-value
		Mean	SD	Mean	SD	
Improves our performance	289	3.72	1.24	4.11	1.04	< 0.001
Increases our productivity	284	3.93	1.21	4.27	1.02	< 0.001
Enhances our effectiveness	285	3.68	1.25	4.02	1.12	< 0.001
Is useful	285	4.06	1.13	4.39	0.93	< 0.001
Was easy to learn to use	283	3.97	1.10	4.00	1.07	0.632
Does not require a lot of effort to offer to patients	286	3.84	1.22	3.79	1.22	0.541
We find it easy to use	282	3.99	1.12	4.06	1.04	0.229
I find it easy to get it to do what I want	281	3.75	1.23	3.91	1.14	0.015

Scale from 1 (strongly disagree) to 5 (strongly agree)

Respondents only received this question if they indicated that they provided at least one service via telephone

Due to some missing data, the total Ns per item ranges from 281 to 289

There were also generally positive ratings of the current supports for telehealth in respondents’ organizations for both telephonic and video-based counseling services (Table 4). Average responses for telephonic counseling support items fell between 3.54 and 4.43 (on a scale from 1 to 5) while average responses for video-based counseling support items fell between 3.83 and 4.43 (on a scale from 1 to 5). Overall, views were generally more positive toward video-based counseling, with the exception of questions regarding ease of use and ability to access these technologies, where respondents rated telephonic counseling significantly higher (*ps* < *0.001*). Regarding funding and reimbursement for service provision, respondents indicated significantly greater agreement with being adequately reimbursed and being able to continue to bill for services provided via video than for those provided by telephone (*ps* < *0.001*).

**Table 4:** Comparisons between available supports for telephonic and video counseling

	Number	Telephone counseling		Video counseling		*p*
		*M*	*SD*	*M*	*SD*	
Staff has been properly trained in *(telephone/video counseling)*	197	3.83	1.12	4.03	1.06	< 0.001 (V)
Staff want the use of *(telephone/video counseling)* to be sustained	198	4.20	1.05	4.43	0.93	< 0.001 (V)
*(Telephone/video counseling) i*s affordable to patients	197	4.43	0.87	4.37	0.90	0.275 (T)
Patients find *(telephone/video counseling)* easy to use	198	4.34	0.87	4.01	0.99	< 0.001 (T)
Most of our patients can access *(telephone/video counseling)* technology	201	4.39	0.89	3.87	1.16	< 0.001 (T)
Patients want *(telephone/video counseling)* to be sustained	199	4.32	0.91	4.33	0.97	0.861
Our leadership supports the implementation of *(telephone/video counseling)*	194	4.25	0.98	4.42	0.91	0.004 (V)
Staff, facilities, equipment, job descriptions, and policies are in place to sustain *(telephone/video counseling)*	194	4.13	1.05	4.25	0.96	0.061
There is a clinical champion for the promotion of *(telephone/video counseling)* long term	196	3.54	1.33	3.94	1.18	< 0.001 (V)
*(Telephone/video counseling)* easily integrates into our workflow	198	4.24	0.99	4.38	0.91	0.014 (V)
We have the information technology expertise to support the use of *(telephone/video counseling)* in our organization	199	4.06	1.15	4.18	1.13	0.024 (V)
We have the billing expertise to support the use of *(telephone/video counseling)* in our organization	198	4.11	1.06	4.25	0.99	0.005 (V)
We are adequately reimbursed for the services we provide with *(telephone/video counseling)*	198	3.76	1.14	4.04	1.09	< 0.001 (V)
We anticipate continuing to be adequately reimbursed for the services we provide with *(telephone/video counseling)* in the future	192	3.68	1.15	4.01	1.14	< 0.001 (V)
*(Telephone/video counseling)* has reduced racial inequities	194	3.80	1.09	3.83	1.13	0.528
*(Telephone/video counseling)* has reduced the stigma associated with receiving mental health services	194	4.04	1.04	4.12	1.01	0.079
We have been trained in effective delivery of evidence-based practices via *(telephone/video counseling)*	198	3.75	1.21	3.98	1.15	< 0.001 (V)

Scale from 1 (strongly disagree) to 5 (strongly agree)

*N* ranged from 206 to 214 for telephone counseling question responses and *N* ranged from 236 to 246 for video counseling question responses. Because dependent samples *t*-tests were conducted, missing data was deleted pairwise, leaving total Ns ranging from 192 to 201

*V* indicates video was significantly higher; *T* indicates telephone was significantly higher

## Discussion

As the healthcare landscape continues to shift in the wake of the COVID-19 pandemic, questions remain around the role of telehealth in behavioral healthcare moving forward. As healthcare organizations move into a new era of providing care that will now be shaped by their experiences during the pandemic and the knowledge they gained during this time, understanding attitudes and needs surrounding telehealth is vital to being able to continue to provide it effectively. While this time brings new opportunities to adapt and meet patient needs in new ways, it will also bring new challenges, prompting the need to better understand the desires of healthcare providers right now.

There appears to be enormous variation in the amount and type of telehealth and virtual care that healthcare organizations provide, driven by a number of factors, including location, leadership acceptance, reimbursement, technological know-how, and other variables that may still be unexplored. While 85% of respondents said that they provided at least one telehealth service, the number and type of services varied significantly. Among the individuals providing telehealth services, the services most commonly utilized were basic telephone- or video-based counseling. Given the need for these services during the pandemic in order to reach clients, this is understandable. However, despite the high rates of telehealth counseling, few organizations in this study were using phone or video counseling to provide additional services like medications or MAT, despite the increased efficiency and benefits for providers and patients if they were not required to receive these services in-person. These results may reflect a lack of provider comfort with providing and monitoring these services via telehealth, along with regulatory issues.^13–15^

Interestingly, only approximately 50% of respondents indicated that they were using services like text message reminders for appointments or computerized screenings, which are areas that could be easily expanded and would likely have a high impact on reducing no-shows to appointments and lessening workloads.^28,29^ While there is significant interest in the use of text reminders and feedback to increase adherence to treatment plans, improve appointment attendance, and provide more accessible intervention options, relatively little research has assessed the prevalence of these services in healthcare organizations. Anecdotally, it is thought that rates of use for these services might be quite high but current research suggests that roughly 35–37% of healthcare organizations send text reminders about appointments, while about 60% use these services to remind patients about taking medication or other treatment steps.^30,31^ Rates of using this service in our sample are fairly similar to those seen in previous research, indicating that while this is an area that can be useful, there may be barriers to expanding it further in some organizations.^32,33^ This may stem from the cost of implementing this type of service in smaller organizations, lack of knowledge of how to implement this type of technology, and/or concerns around data privacy when using this technology. However, this is an area of emerging interest across healthcare fields and shows great promise to improve treatment adherence and minimize no-shows, benefitting both patients and healthcare organizations.^32,33^

It is notable that every type of service was most commonly offered in-person, though this difference between modalities was not always significant. While it is not possible to assess whether that is due to provider or patient preference with the current study, it appears that there is still utility to and preferences for in-person services. For many services, the number of respondents indicating they offered this service by video or telephone was not significantly different but in-person was significantly higher. The uncertainty around payment and reimbursement structures for telehealth services and the ongoing rollback of COVID-19 era policies that allowed for more flexible delivery of behavioral health services may help to explain some of these differences. Without appropriate licensing, reimbursement, and privacy policies to support behavioral healthcare providers, many organizations may simply find it easier to continue providing services by traditional in-person methods, helping to account for the higher levels of in-person care.^34,35^ Additionally, because many of these are services that are extremely difficult to provide without an in-person component, such as partial hospitalization or in-home supports, this may help to explain the discrepancy. It is notable that while some respondents indicated providing particular these services via telehealth, it may not be possible to provide such services via telehealth, and such, these answers may not be completely interpretable given this context.

However, other services, like case management and peer support, could be facilitated by telehealth relatively easily, but these results may suggest that organizations see value in providing these services in-person above and beyond doing so only virtually. Lastly, our finding that there was not a significant difference in how frequently respondents indicated that their organizations were providing individual therapy in-person versus by video suggests the durability of telehealth, at least for this type of treatment. Importantly, this study did not examine whether respondents did not provide a service at all and consider that in these comparisons which may be a path for future research. While telehealth services might not be right for every behavioral health service, it is clear that they can be delivered effectively for some services and that, even in these cases, a preference for video over only telephone-based care exists.

In a different area of services, only roughly 50% of respondents indicated that their organizations use mobile apps for any type of treatment or support purposes. For many individuals, mobile apps can be a useful treatment tool, either as an adjunct to other forms of treatment or as an additional form of support between treatment sessions. Recovery and other supportive mobile apps can help individuals in treatment form a community, get support when they need it, and provide resources as they navigate transitions out of more intensive treatment settings.^35^ However, the use of mobile apps in treatment is still relatively new and many providers may not have significant experience with doing so and may feel uncomfortable. Moreover, some clients may not be able or willing to utilize mobile apps, even if their provider would like them to, potentially limiting the reach of apps in certain populations.^36^ Despite all this, the use of mobile apps is a burgeoning area in mental health treatment and an area that many treatment organizations may want to consider emphasizing in this new post-pandemic phase.

The most commonly used telehealth services in respondents’ organizations, telephone- and video-based counseling, were well-regarded but respondents identified potential areas for improvement. Overall, opinions of both types of telehealth were quite positive, with some preference for video-based counseling. It is possible that some variation in preference for one type of telehealth may relate to the location or client populations served. Providers who work primarily with older adults or individuals in rural areas with limited technology access may prefer telephone-based counseling because that is what their clients prefer. It is not possible to know how much of the variation these variables explain, though, based on the available data. However, in general, there still remains an overall staff preference for video-based over telephone-based care. The authors cannot report on patient preferences since the patient perspective was not part of this research.

Additionally, though providers and organizations have developed the necessary support and expertise to utilize telehealth care during the pandemic, further work is necessary to sustain these changes long term. Some concerns, such as feelings of inadequate training on the use of such technologies, are relatively easy to address.^37^ Other concerns may require more in-depth effort to be resolved, such as the need for greater organizational support for the implementation of such technologies, the development of greater technological expertise, both on a provider and organizational level, to support the use of telehealth technologies, and increasing patient access and technological ability.^38,39^ While these may require more effort, they are resolvable if organizations have the desire to continue to grow their use of telehealth and ensure that more patients are able to access such services.

As it becomes clear that both providers and patients have found value in the use of telehealth care, ensuring that organizations and providers are equipped to effectively provide these services will support their provision in the future. Though shifts in the healthcare landscape as a result of the pandemic have facilitated the provision of telehealth services, challenges persist, and further work will be required in order to ensure that telehealth will remain a viable service. Facilitating needed support from organizational leadership and others with experience in the provision of telehealth services will allow for continued effective service provision. However, larger shifts including promoting greater access to technology, for both patients and organizations, and developing stronger understandings of how to use these technologies will allow greater use. Moreover, providing training to organizations and providers on how to provide particular types of care via telehealth, such as medication management, is particularly vital to increasing access to care for populations that may struggle to access services, but these services are challenging to provide online and may require additional training.

### Limitations

This study had a relatively small sample size, though the sample was geographically diverse (respondents came from 43 states and Puerto Rico) and represented a diversity of organizational types and job roles, which is a strength of the survey. Given the method by which surveys were distributed, the exact response rate is unknown. However, these distribution methods were also a strength as they allowed for an efficient, diverse geographic distribution and promoted a wide range of respondents. Other limitations may be that respondents to this survey may be those who are more likely to be using telehealth and, therefore, want to respond to a survey of this nature, so their opinions may not be generalizable to the larger behavioral health field.

### Conclusion

After the rapid increase of telehealth use during the COVID-19 pandemic, telehealth remains quite popular, particularly for behavioral healthcare needs. Though telehealth cannot meet the needs of every patient or provider, it shows great promise to increase access to services and allow providers to serve their communities more efficiently. As it is now clear that telehealth will remain a part of the healthcare landscape for the foreseeable future, understanding the current state of telehealth in behavioral health organizations and where organizations may be struggling will allow organizations to plan future improvements. Focusing development on areas that will allow organizations to serve their clients most effectively and efficiently will be vital to improving access to care when these services are in particularly high demand.

## Implications for Behavioral Health

Telehealth continues to be a convenient and affordable way to improve access to healthcare, but as regulations, policies, and provider and patient attitudes around its use continue to shift, understanding these views is vital to expanding its use throughout the behavioral healthcare system. This study demonstrates that interest in and use of telehealth for many services in the provision of behavioral health services is quite high, particularly for those that can be delivered via video-based platforms. However, there are also many areas where services could be expanded, such as the use of text messaging and mobile apps in treatment, with minimal cost and effort, and where the impact could be significant. Further, these results also outline the value of in-person services, even when telehealth use could be possible. For services like peer support or case management, many organizations continue to prefer the use of in-person services even if it is possible to provide these services via telehealth, suggesting the value of balancing both in-person and telehealth services in behavioral health organizations. Moving forward, these results suggest that it will be important for organizations to consider where it may be possible to expand their provision of telehealth services, particularly in areas that would not require significant investments of time or effort to do so, while also considering areas in which the benefit of in-person care may supersede the desire to utilize telehealth.

## Appendix 1. Survey measures

**Table:**

Measure type	Answer choices:
Demographics	
Organizational location	• Urban • Suburban • Small city • Rural • Tribal reservation
Tribal or indigenous serving	• Yes/no
Organizational type	• Health system • Federally qualified healthcare center • Specialty behavioral health provider: 6 + sites • Specialty behavioral health provider: 2–5 sites • Specialty behavioral health provider: stand-alone • Private practice
Organizational role	• Administrator • Clinical supervisor • Counselor • Social worker • Psychologist • Physician/prescriber • Nurse • Case worker • Peer support worker • Other
Provision of telehealth measures	
Currently technologies in use in organization (yes/no)	• Computerized screenings/assessments • Text appointment reminders • Texts to solicit patient experience feedback • Text motivational messages • Organizational web portal for patients to use to access services • Video-based counseling • Video-based therapy to provide psychiatric medicines • Video-based therapy to provide medication-assisted treatment (MAT) • Mobile app(s) during treatment • Mobile app(s) during post-treatment recovery • Secure chats for recovery support sessions • Telephone-based counseling • Telephone-based recovery supports • Virtual worlds for treatment
Use of telehealth services (in-person/telephone-based services/video-based services)	• Screening and assessment • Individual therapy • Group therapy • Medication management • Therapy services during partial hospitalization • Therapy sessions during residential treatment • Peer supports • Case management • Multi-disciplinary team-based services (e.g., Assertive Community Treatment) • In-home supports • Psychoeducation • Employment supports • Education supports
Organizational readiness for technology use (strongly disagree(1)-strongly agree(5))	
	• Staff has been properly trained in the use of this technology • Staff wants the use of this technology the be sustained • This technology is affordable for patients • Patient support • Patient accessibility • Leadership supports the implementation of this technology • Having staff, facilities, and equipment in place to support the technology • Having a clinical champion for the technology • The ease of integrating the technology into workflow • Availability of information technology experts to support use of the technology • Adequate reimbursement for the use of this technology • Billing expertise for the technology • Potential for this technology to increase accessibility and reduce stigma associated with seeking care • These technologies help to promote racial equity • Staff are effectively trained to provide evidence-based treatment via these technologies
Technology acceptance (strongly disagree (1)-strongly agree(5))	
	*Ease-of-use scale* • Was easy to learn • Offering this technology does not require a lot of effort • Is easy to use • It is easy to get this technology to do what I want *Perceived usefulness scale* • Improves our performance • Increases our productivity • Enhances our effectiveness • Is useful
